# Pembrolizumab versus chemotherapy in recurrent, advanced urothelial cancer in Japanese patients: a subgroup analysis of the phase 3 KEYNOTE-045 trial

**DOI:** 10.1007/s10147-019-01545-4

**Published:** 2019-11-15

**Authors:** Hiroyuki Nishiyama, Yoshiaki Yamamoto, Naoto Sassa, Kazuo Nishimura, Kiyohide Fujimoto, Satoshi Fukasawa, Minato Yokoyama, Hideki Enokida, Kenichi Takahashi, Yoshinobu Tanaka, Kentaro Imai, Takashi Shimamoto, Rodolfo Perini, Tara Frenkl, Dean Bajorin, Joaquim Bellmunt

**Affiliations:** 1 University of Tsukuba Hospital, 2 Chome-1-1 Amakubo, Tsukuba, Ibaraki 305-8576 Japan; 2 Yamaguchi University Hospital, 1 Chome-1-1 Minamikogush, Ube, Japan; 3 Nagoya University Hospital, 65 Tsurumaicho, Showa Ward, Nagoya, Japan; 4 Osaka International Cancer Institute, 3 Chome-1-6 9 Otemae, Chuo Ward, Osaka, Japan; 5 Nara Medical University Hospital, 840 Shijocho, Kashihara, Japan; 6 Chiba Cancer Center, 666-2 Nitonacho, Chiba, Japan; 7 Medical Hospital, Tokyo Medical and Dental University, 1 Chome-5-45 Yushima, Tokyo, Japan; 8 Kagoshima University Hospital, 8-35-1 Sakuragaoka, Kagoshima, Japan; 9 MSD K.K., Kitanomaru Square, 1-13-12, Kudan Kita, Tokyo, Japan; 10 Merck & Co., Inc., 2000 Galloping Hill Rd, Kenilworth, NJ USA; 11 Memorial Sloan Kettering Cancer Center, 1275 York Avenue, New York, NY USA; 12 PSMAR-IMIM Research Institute, Carrer del Dr. Aiguader, 88, Barcelona, Spain; 13 Harvard Medical School, 25 Shattuck St, Boston, MA USA

**Keywords:** Pembrolizumab, Advanced urothelial cancer, Japanese, KEYNOTE-045, Immune checkpoint blockade

## Abstract

**Background:**

The open-label, randomized, active-controlled KEYNOTE-045 study (NCT02256436) showed that second-line pembrolizumab significantly improved overall survival (OS) of patients with advanced/metastatic urothelial cancer (UC) that progressed after first-line platinum-containing chemotherapy, compared with standard chemotherapy (paclitaxel, docetaxel, or vinflunine). Pembrolizumab is approved for patients with bladder cancer in Japan.

**Patients and methods:**

Analysis was performed in the subgroup of Japanese patients enrolled in the KEYNOTE-045 study. Coprimary end points were OS and progression-free survival (PFS). Objective response rate (ORR) and safety were secondary end points.

**Results:**

Fifty-two Japanese patients (pembrolizumab, *n* = 30; chemotherapy, *n* = 22) were followed up for a median of 26.1 months. Patients who received pembrolizumab compared with chemotherapy had a 19% lower risk for death (hazard ratio [HR] 0.81, 95% CI 0.44–1.50); after adjusting for baseline covariates, the HR for OS was 0.61 (95% CI 0.32–1.15). The 24-month OS rate was higher with pembrolizumab (26.9% vs 14.3%). PFS was 2.0 and 4.9 months for pembrolizumab and chemotherapy, respectively (HR 1.71, 95% CI 0.95–3.08). ORR was similar for pembrolizumab and chemotherapy (20.0% vs 18.2%); durability of response was higher with pembrolizumab: 67% and 33% of patients, respectively, maintained a response for > 12 months. Treatment-related adverse events, including grade 3–5 events, occurred less frequently with pembrolizumab.

**Conclusions:**

Pembrolizumab provided durable antitumor activity in patients with locally advanced/metastatic UC that progressed after platinum-containing chemotherapy in the overall population and in the Japanese subgroup; safety profile was consistent with that previously observed for pembrolizumab.

**Electronic supplementary material:**

The online version of this article (10.1007/s10147-019-01545-4) contains supplementary material, which is available to authorized users.

## Introduction

Urothelial cancers (UCs) include a variety of tumors from the urinary tract, renal pelvis, ureter, and urethra, but > 90% of all UCs originate in the bladder [1]. Bladder cancer is the tenth most common cancer worldwide, accounting for approximately 550,000 new cancer diagnoses annually, and the sixth most common cancer in men [2]. In Japan, bladder cancer is the 13th most common cancer overall, and the eighth most common cancer in men [3]. Although the incidence of bladder cancer is lower in Asia than in Western countries [2], its incidence in Japan (5.6 per 100,000) is among the highest in Asia [3, 4]. This might be because smoking is a major risk factor for bladder cancer; however, the prevalence of smoking among Japanese men decreased from 84% in 1965 to 30% in 2014 [5]. It was expected that more than 20,000 cases would be diagnosed in 2018, with approximately three-quarters of those cases in men [6]. The mortality rate for Japanese patients is 25%, and 5-year survival rates for patients diagnosed with bladder cancer are 76.5% in men and 64.4% in women [4, 7].

Development of new antitumor agents for metastatic bladder cancer is an urgent priority in Japan [7]. In 2009, the Japanese Urological Association issued guidelines for first-line treatment of bladder cancer, recommending methotrexate, vinblastine, doxorubicin, and cisplatin or gemcitabine plus cisplatin as systemic chemotherapy for metastatic bladder cancer, because these compounds have showed comparable efficacy in clinical trials [8]. However, the gemcitabine plus cisplatin regimen is preferred because of a more favorable tolerability profile [8]. No recommendation has been made regarding second-line treatment or treatment of patients ineligible for platinum-based therapy, and a variety of combination and single-agent treatments are used in the management of UC in clinical practice in Japan [8–10].

Bladder cancer is associated with a high mutation burden and is highly antigenic, which has been correlated with higher immunotherapy response rates in UC and in other cancers, such as melanoma and lung cancer [11]. The potential of immunotherapy in bladder cancer treatment has been known for approximately 30 years, since the efficacy of using intravesical bacillus Calmette–Guérin to provoke an immune response to treat certain types of bladder cancer was identified [11]. Programmed death ligand 1 (PD-L1) expression on tumor cells can suppress immune responses against tumors by binding to programmed death 1 (PD-1) receptors on T cells [11, 12]. Monoclonal antibodies against this pathway have been developed to promote antitumor immune responses in patients with advanced or metastatic cancers; results of phase 1/2 studies showed that monoclonal antibodies have promising efficacy and manageable tolerability in management of UC [13–17]. As a result, 3 anti–PD-L1 and 2 anti–PD-1 antibodies have been approved by the US Food and Drug Administration for the treatment of patients with platinum-refractory bladder cancer [11, 12].

The randomized phase 3 KEYNOTE-045 study is the only study that shows an overall survival (OS) benefit with pembrolizumab compared with chemotherapy. Use of pembrolizumab (vs chemotherapy) significantly improved OS and objective response rates (ORR) of patients with recurrent, advanced UC that progressed during or after platinum-based chemotherapy [18]. This outcome was consistently observed independent of PD-L1 expression, presence of liver metastases, or type of chemotherapy comparator, and it has been confirmed after more than 2 years of follow-up [18, 19]. Compared with chemotherapy, pembrolizumab had a better safety profile and was associated with improved quality-of-life outcomes [18–20].

Pembrolizumab is currently the only immune checkpoint inhibitor approved in Japan for treating platinum-refractory UC [12]. A subgroup analysis of the KEYNOTE-045 study was performed to compare pembrolizumab with chemotherapy in Japanese patients with progressive UC. The results of this subgroup analysis are presented herein.

## Patients and methods

### Study design and patient population

A full description of the KEYNOTE-045 study has been published [18, 19]. Patients included in this subgroup analysis were enrolled in 20 study centers in Japan and had been diagnosed with transitional cell–predominant UC of the bladder, renal pelvis, ureter, or urethra, and experienced progressive disease after first- or second-line platinum-based chemotherapy or recurrence < 12 months after perioperative platinum-based chemotherapy. Patients had an Eastern Cooperative Group performance status (ECOG PS) of 0, 1, or 2 and a tumor sample available for biomarker assessment.

This study was conducted in accordance with Good Clinical Practice guidelines and the Declaration of Helsinki, and the study protocol was approved by institutional review boards or ethics committees of all participating sites. All patients provided written informed consent to participate before enrollment, and the study was registered on ClinicalTrials.gov before enrollment of the first patient (ClinicalTrials.gov, NCT02256436).

### Treatment and assessments

Patients were randomly assigned 1:1 to receive either intravenous (IV) pembrolizumab 200 mg every 3 weeks (Q3W) or investigator’s choice of paclitaxel (175 mg/m^2^ IV Q3W), docetaxel (75 mg/m^2^ IV Q3W), or vinflunine (320 mg/m^2^ IV Q3W). However, vinflunine was not an approved second-line treatment for bladder cancer in Japan at the time of this study; therefore, none of the patients in Japan received that treatment. Patients were stratified by ECOG PS score (0/1 vs 2), presence of liver metastases (yes vs no), hemoglobin concentration (< 10 g/dL vs ≥ 10 g/dL), and time since the last dose of chemotherapy (< 3 vs ≥ 3 months) before randomization. Patients were treated until disease progression, unacceptable toxicity, patient or investigator decision to discontinue treatment, or completion of 2 years of pembrolizumab treatment, whichever occurred first.

Tumor response was assessed by blinded, independent, central radiologic review per Response Evaluation Criteria in Solid Tumors (RECIST) v1.1 [21]. The full assessment schedule is provided elsewhere [18]. Adverse events (AEs) were assessed and graded per National Cancer Institute Common Terminology Criteria for Adverse Events, version 4.0 [22]. All treatment-emergent AEs were evaluated for potential immunologic etiology.

Patients were followed up for at least 30 days after the last dose of study treatment or until initiation of a new line of treatment. Radiologic imaging for disease status continued per study post-treatment follow-up schedule for patients who discontinued therapy without documented disease progression.

### Study end points

Study end points have been previously published [18, 19]. Coprimary end points included OS and progression-free survival (PFS). Additional coprimary end points assessed OS and PFS for patients with PD-L1–positive tumors (combined positive score [CPS] ≥ 10). Key secondary end points included ORR, duration of response (DOR), and safety and tolerability. Prespecified health-related quality-of-life (HRQOL) analysis was conducted using end points of time to traditional deterioration (TTD), defined as time to first onset of a ≥ 10-point decrease from baseline without confirmation under right-censoring rule (the last observation) and change from baseline in the European Organisation for Research and Treatment of Cancer Quality of Life Questionnaire Core 30 (EORTC QLQ-C30) global health status/QOL score at week 15.

### Statistical analyses

Primary efficacy end points and safety end points were analyzed using data from the intention-to-treat (ITT) population and all-patients-as-treated population (all randomly assigned patients who received at least 1 dose of study treatment), respectively. Median OS and PFS were estimated using the Kaplan–Meier method. Multivariate Cox proportional hazards models were used to adjust the hazard ratio (HR) for OS, taking into consideration the following baseline covariates: ECOG PS (0 vs 1/2), visceral metastases (presence vs absence), hemoglobin level (≥ 10 g/dL vs < 10 g/dL), and time from completion of most recent chemotherapy (< 3 months vs ≥ 3 months). Median DOR was summarized descriptively using Kaplan–Meier analysis of the subset of patients who experience complete response or partial response. Additional details of the statistical analyses have been described previously [18, 19]. The stratified Cox model was used and the log-rank test was performed in the overall population; however, corresponding unstratified analyses were performed in the Japanese population because of the small sample size.

Change in baseline EORTC QLQ-C30 score at week 15 was assessed using pairwise comparisons. HRs for TTD were calculated using a Cox regression model with treatment as a covariate.

## Results

### Patients

Enrolled Japanese patients (*n* = 52) were randomly assigned to receive either pembrolizumab (*n* = 30) or chemotherapy (*n* = 22; paclitaxel, *n* = 11; docetaxel, *n* = 11) (see Supplementary Fig. 1). Key differences in baseline characteristics between the pembrolizumab arm and the chemotherapy arm were the percentages of patients with lymph-node metastases only (17.7% vs 36.4%) or visceral metastases (83.3% vs 63.6%) at baseline (Table 1). Twelve patients had tumors with PD-L1 CPS ≥ 10 (pembrolizumab, *n* = 4; chemotherapy, *n* = 8). The low number of patients and the imbalance between the arms precluded the inclusion of any meaningful analysis based on PD-L1 expression.

**Table 1:** Patient baseline characteristics

Characteristic	Pembrolizumab *N* = 30	Chemotherapy *N* = 22
Age (years), median (range)	72 (51–83)	70.5 (56–84)
Sex, *n* (%)		
Male	24 (80.0)	16 (72.7)
Geographic location of disease, *n* (%)		
Upper tract	12 (40.0)	5 (22.7)
Lower tract	18 (60.0)	17 (77.3)
ECOG PS, *n* (%)		
0	23 (76.7)	14 (63.6)
1	7 (23.3)	7 (31.8)
2	0 (0)	1 (4.5)
Visceral disease, *n* (%)	25 (83.3)	14 (63.6)
Disease in lymph node only, *n* (%)	5 (17.7)	8 (36.4)
Liver metastases, *n* (%)	7 (23.3)	4 (18.2)
Hemoglobin < 10 g/dL,^a^*n* (%)	8 (26.7)	4 (18.2)
Time since completion of most recent prior therapy, *n* (%)		
≥ 3 months	17 (56.7)	10 (45.5)
< 3 months	13 (43.3)	12 (54.5)
Setting of most recent prior therapy, *n* (%)		
Neoadjuvant	1 (3.3)	2 (9.1)
Adjuvant	1 (3.3)	4 (18.2)
First line	24 (80.0)	12 (54.5)
Second line	4 (13.3)	4 (18.2)
Prior platinum-based therapy, *n* (%)		
Cisplatin	24 (80.0)	18 (81.8)
Carboplatin	6 (20.0)	2 (9.1)
Other^b^	0 (0)	2 (9.1)
Smoking status, *n* (%)		
Never	13 (43.3)	8 (36.4)
Former	15 (50.0)	12 (54.5)
Current	2 (6.7)	2 (9.1)
PD-L1 CPS ≥ 10, *n* (%)	4 (13.3)	8 (36.4)
Risk factors,^c^*n* (%)		
0	8 (26.7)	4 (18.2)
1	13 (43.3)	10 (45.5)
2	5 (16.7)	6 (27.3)
3 or 4	4 (13.3)	2 (9.1)
EORTC QLQ-C30 global health status/QOL score	57.8 (25.4)	54.9 (27.4)

*CPS* combined positive score, *ECOG PS* Eastern Cooperative Oncology Group performance status, *EORTC QLQ-C30* European Organisation for Research and Treatment of Cancer Quality of Life Questionnaire Core 30, *PD-L1* programmed death ligand-1, *QOL* quality of life

^a^Latest hemoglobin test value before or on randomization date

^b^Oxaliplatin, nedaplatin

^c^Bellmunt risk factors of ECOG PS > 0, hemoglobin level < 10 g/dL, and liver metastases [31] + time from prior chemotherapy < 3 months [32]

Median follow-up was 26.1 months (range 0.9–29.5 months) at data cutoff on October 26, 2017. At the time of analysis, all patients administered chemotherapy and 28 (93.3%) patients administered pembrolizumab had discontinued treatment. Two patients had completed the maximum 2 years of pembrolizumab treatment. Median (range) duration of treatment was 3.04 months (1 day–23.92 months) for pembrolizumab and 1.76 months (1 day–24.44 months) for chemotherapy.

### Efficacy

**Fig. 1:**
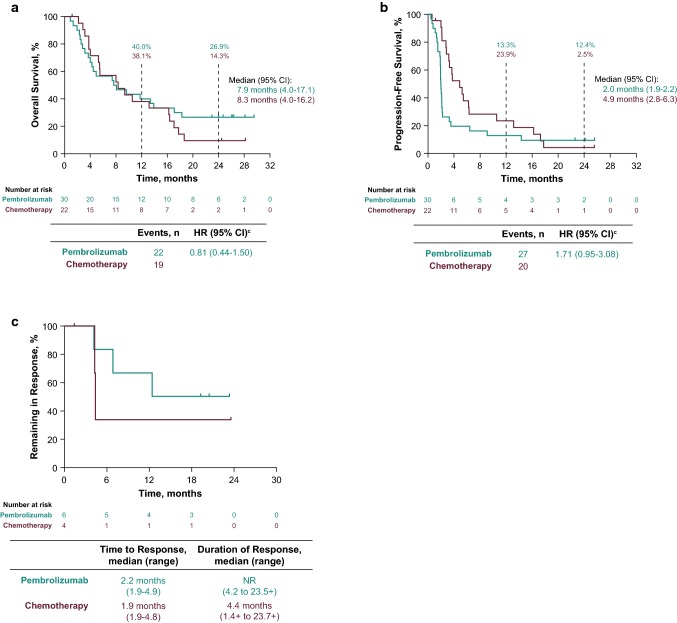
Kaplan–Meier estimates of **a** OS, **b** PFS, and **c** DOR^a^ in Japanese patients (ITT population) with advanced or metastatic urothelial carcinoma treated with pembrolizumab (*n* = 30) or chemotherapy (*n* = 22). ^a^DOR was assessed in patients who achieved an objective response of CR or PR. *CI* confidence interval, *CR* complete response, *DOR* duration of response, *HR* hazard ratio, *ITT* intention-to-treat, *NR* not reached, *OS* overall survival, *PFS* progression-free survival, *PR* partial response

Median OS was 7.9 months (95% CI 4.0–17.1) with pembrolizumab and 8.3 months (95% CI 4.0–16.2) with chemotherapy (HR 0.81, 95% CI 0.44–1.50, Fig. 1a); patients who received pembrolizumab had a 19% lower risk for death than patients who received chemotherapy. Results of multivariate analysis showed that the HR for OS was 0.61 (95% CI 0.32–1.15) after adjusting for additional covariates. OS at 6 and 12 months was comparable between patient groups; 24-month OS rates were higher in the pembrolizumab group (26.9%) than in the chemotherapy group (14.3%). Median PFS was longer in the chemotherapy arm than in the pembrolizumab arm (4.9 months [95% CI 2.8–6.3] and 2.0 months [95% CI 1.9–2.2], respectively; HR 1.71, 95% CI 0.95–3.08, Fig. 1b).

ORRs for pembrolizumab and chemotherapy were similar (20.0% vs 18.2%; Table 2). The disease control rate was 26.7% (stable disease, 6.7%) in the pembrolizumab arm and 63.6% (stable disease, 45.5%) in the chemotherapy arm. At data cutoff, median DOR had not been reached (range 4.2 to 23.5+ months) for responders in the pembrolizumab arm and was 4.4 months (range 1.4+ to 23.7+ months) for responders in the chemotherapy arm (Fig. 1c). Responses were maintained for at least 12 months by 67% of responders to pembrolizumab therapy and by 33% of responders to chemotherapy.

**Table 2:** Best overall response (ITT population)

Best overall response	Pembrolizumab *N* = 30		Chemotherapy *N* = 22	
	*n*	% (95% CI)^a^	*n*	% (95% CI)^a^
Objective response rate (CR + PR)	6	20.0 (7.7–38.6)	4	18.2 (5.2–40.3)
CR	3	10.0 (2.1–26.5)	1	4.5 (0.1–22.8)
PR	3	10.0 (2.1–26.5)	3	13.6 (2.9–34.9)
SD	2	6.7 (0.8–22.1)	10	45.5 (24.4–67.8)
Disease control rate (CR + PR + SD)	8	26.7 (12.3–45.9)	14	63.6 (40.7–82.8)
Progressive disease	20	66.7 (47.2–82.7)	3	13.6 (2.9–34.9)
No assessment	0	6.7 (0.8–22.1)	3	9.1 (1.1–29.2)
Nonevaluable	2	0.0 (0.0–11.6)	2	13.6 (2.9–34.9)

*CI* confidence interval, *CR* complete response, *ITT* intention-to-treat, *PR* partial response, *SD* stable disease

^a^Based on binomial exact CI method

### Safety

Treatment-related AEs (TRAEs) of any grade and of grade 3–5 were reported in 56.7% and 16.7%, respectively, of patients in the pembrolizumab arm and in 95.5% and 77.3%, respectively, of patients in the chemotherapy arm. Three patients in the pembrolizumab arm (interstitial lung disease [*n* = 2]; pneumonitis [*n* = 1]) and 2 patients in the chemotherapy arm (interstitial lung disease [*n* = 1]; cardiac failure [*n* = 1]) discontinued treatment because of TRAEs.

TRAEs that occurred in > 15% of patients in the chemotherapy arm were alopecia, decreased white blood cell count, decreased appetite, decreased neutrophil count, nausea, peripheral sensory neuropathy, anemia, neutropenia, fatigue, malaise, stomatitis, diarrhea, and dysgeusia (Table 3). The most common TRAEs in the pembrolizumab arm were pruritus (*n* = 6 [20.0%] patients), hypothyroidism (*n* = 4 [13.3%]), rash (*n* = 4 [13.3%]), nausea (*n* = 3 [10.0%]), and malaise (*n* = 3 [10.0%]).

**Table 3:** TRAEs of any grade and grade 3 or 4 occurring in > 5% of patients in either treatment group (APaT population)

TRAEs, *n* (%)	Pembrolizumab *N* = 30		Chemotherapy *N* = 22	
	Any grade	Grade 3–5	Any grade	Grade 3–5
Any	17 (56.7)	5 (16.7)	21 (95.5)	17 (77.3)
Alopecia	–	–	12 (54.5)	–
Decreased white blood cell count	–	–	9 (40.9)	8 (36.4)
Decreased appetite	1 (3.3)		9 (40.9)	1 (4.5)
Decreased neutrophil count	–	–	8 (36.4)	7 (31.8)
Nausea	3 (10.0)	–	8 (36.4)	–
Peripheral sensory neuropathy	–	–	8 (36.4)	–
Anemia	–	–	7 (31.8)	4 (18.2)
Neutropenia	–	–	7 (31.8)	6 (27.3)
Fatigue	2 (6.7)	–	8 (36.4)	2 (9.1)
Malaise	3 (10.0)	–	5 (22.7)	–
Pruritus	6 (20.0)	–	2 (9.1)	–
Stomatitis	1 (3.3)	1 (3.3)	4 (18.2)	–
Diarrhea	1 (3.3)	–	4 (18.2)	–
Dysgeusia	–	–	4 (18.2)	–
Decreased lymphocyte count	1 (3.3)	1 (3.3)	3 (13.6)	3 (13.6)
Hiccups	–	–	3 (13.6)	–
Rash	4 (13.3)	1 (3.3)	1 (4.5)	–
Hypothyroidism	4 (13.3)	–	–	–
Upper abdominal pain	1 (3.3)	–	2 (9.1)	–
Febrile neutropenia	–	–	2 (9.1)	2 (9.1)
Hyperkalemia	–	–	2 (9.1)	2 (9.1)
Hyponatremia	–	–	2 (9.1)	2 (9.1)
Leukopenia	–	–	2 (9.1)	2 (9.1)
Nail discoloration	–	–	2 (9.1)	–
Constipation	–	–	2 (9.1)	–
Peripheral edema	–	–	2 (9.1)	–
Interstitial lung disease	2 (6.7)	1 (3.3)	1 (4.5)	–
Hyperthyroidism	2 (6.7)	–	–	–
Pyrexia	2 (6.7)	–	1 (4.5)	–

*APaT* all patients as treated, *TRAE* treatment-related adverse event

Seven treatment-related serious AEs (SAEs) were reported among 5 (16.7%) patients administered pembrolizumab and 2 events were reported in 2 (9.1%) patients administered chemotherapy. No treatment-related SAEs were reported by more than 1 patient, except for interstitial lung disease in 2 patients in the pembrolizumab arm.

12 immune-mediated AEs were reported in 9 (30.0%) patients administered pembrolizumab; the most common were hypothyroidism (*n* = 4), hyperthyroidism (*n* = 2), and interstitial lung disease (*n* = 2). One patient with grade 1 or 2 and 4 patients with grade 3–5 immune-mediated AEs in the pembrolizumab arm received systemic corticosteroids. Single events were also reported for colitis, pneumonitis, rash, and type 1 diabetes. One immune-mediated AE of interstitial lung disease was reported in the chemotherapy arm. No deaths from TRAEs were reported.

### HRQOL

**Fig. 2:**
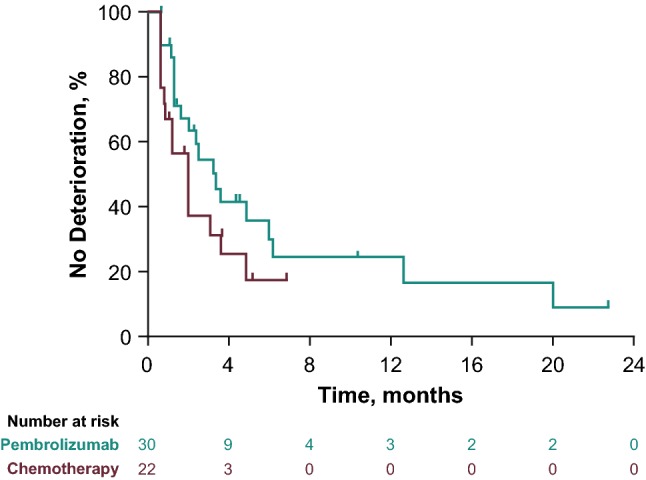
TTD in HRQoL in Japanese patients (ITT population) with advanced or metastatic urothelial carcinoma treated with pembrolizumab (*n* = 30) or chemotherapy (*n* = 22). *HRQoL* health-related quality of life, *ITT* intent-to-treat, *TTD* time to traditional deterioration

No significant difference in EORTC QLQ-C30 scores was observed at week 15 of treatment between the pembrolizumab (*n* = 18) and chemotherapy arms (*n* = 10; least-squares mean change from baseline: + 2.14 vs – 0.66 points). However, a trend toward a delay in TTD was observed for patients randomly assigned to receive pembrolizumab versus chemotherapy (20 vs 15 events; HR, 0.58, 95% CI, 0.29–1.16; Fig. 2).

## Discussion

There is an unmet need for a safe and effective second-line treatment recommendation for Japanese patients with platinum-refractory advanced UC. Current second-line chemotherapy options include single agents docetaxel and paclitaxel; both have been associated with modest activity and hematologic AEs [23]. The current subgroup analysis of KEYNOTE-045 is the first investigation of the safety and efficacy of pembrolizumab in Japanese patients with locally advanced/metastatic UC that progressed after platinum-based chemotherapy compared with standard chemotherapy.

Overall results of the ITT population in the KEYNOTE-045 study showed that pembrolizumab resulted in significantly longer OS—by approximately 3 months—than investigator’s choice of docetaxel or vinflunine (10.1 vs 7.3 months; HR 0.70, 95% CI 0.57–0.85, *p* < 0.001) [18, 19]. The benefit of pembrolizumab compared with chemotherapy was observed in the total population and among patients with tumors that expressed PD-L1 CPS ≥ 10 (HR 0.55, 95% CI 0.37–0.81, *p* = 0.001) [19]. ORR was significantly higher in the pembrolizumab arm than in the chemotherapy arm (21.1 vs 11.0%) [18, 19]. In the overall population, pembrolizumab also had a better safety profile than chemotherapy.

The current analysis was conducted to assess the safety and efficacy of pembrolizumab and chemotherapy among Japanese patients enrolled in the KEYNOTE-045 study. Even with a higher percentage of Japanese patients with visceral disease in the pembrolizumab arm than in the control arm (83.3% vs 63.6%) at baseline, results showed that the risk for death was reduced by 19% in the pembrolizumab arm (HR 0.81). After adjusting for baseline covariates in the multivariate Cox model, the HR for OS improved to 0.61. With additional follow-up, there was a trend toward more favorable median OS in the pembrolizumab arm than in the chemotherapy arm (data not shown), and 24-month OS rates were higher with pembrolizumab (26.9%) than with chemotherapy (14.3%). In the Japanese subgroup, the proportions of patients with objective responses were similar between the pembrolizumab and chemotherapy arms but trended toward a more favorable response with pembrolizumab. At the time of analysis, 67% of patients in the pembrolizumab arm and 33% of patients in the chemotherapy arm maintained a response for > 12 months. Because the presence of visceral disease is a negative prognostic factor for UC, greater proportions of patients with visceral disease in the pembrolizumab may contribute to the longer PFS observed in the chemotherapy arm.

A lack of improvement in PFS with simultaneous improvement in OS was also shown in the ITT population of KEYNOTE-045, which remains the only randomized trial of UC to show a survival benefit of immunotherapy compared with chemotherapy. This pattern of response, OS improvement but not PFS improvement, has also been reported in randomized trials with checkpoint inhibitors in other tumor types, continuing to suggest that PFS may not be a reliable surrogate end point for the clinical benefit of immunotherapy [24–27].

In the current analysis, pembrolizumab was generally well tolerated in Japanese patients and had a better safety profile than chemotherapy. In the KEYNOTE-045 primary analysis population and in the Japanese subgroup, a lower percentage of patients who received pembrolizumab, compared with chemotherapy, experienced grade 3–5 TRAEs (15.0% vs 49.4% in the overall population and 16.7% vs 77.3% in the Japanese subgroup). Consistent with the known safety profile of pembrolizumab, immune-mediated AEs occurred more frequently with pembrolizumab than with chemotherapy. No deaths were attributed to TRAEs.

Pembrolizumab may also result in improved QOL compared with chemotherapy. There was a trend toward a delay in TTD for Japanese patients who received pembrolizumab compared with chemotherapy. This outcome is supported by results of the HRQOL analysis of the overall population enrolled in the KEYNOTE-045 study, which showed that patients who received pembrolizumab had significantly longer TTD than patients who received chemotherapy (*p* = 0.004) [20].

According to the National Comprehensive Cancer Network, pembrolizumab is the preferred second-line therapy for patients with advanced/metastatic bladder cancer and may also be recommended as first-line therapy for patients whose tumors express PD-L1 CPS ≥ 10 or who are ineligible to receive platinum-based therapy [23]. Pembrolizumab is approved for second-line treatment of patients with advanced/metastatic UC in Japan [12]; however, there are no clear guidelines on choosing second-line therapy for these patients. In clinical practice, a variety of combination and single-agent therapies are prescribed [9, 10, 28]. One small study was conducted to evaluate second-line combinations of gemcitabine and taxanes in Japanese patients with metastatic UC that progressed after platinum-based chemotherapy (*N* = 78) [28]. Among 70 evaluable patients treated with gemcitabine combined with either docetaxel or paclitaxel, ORR was 8.6% and median PFS was 3.5 months. Median OS was 9.6 months, with no significant difference in survival between the regimens. The most commonly reported grade ≥ 3 hematologic AEs in the gemcitabine plus paclitaxel arm and the gemcitabine plus docetaxel arm were neutropenia (80.5% vs 59.5%), anemia (43.9% vs 10.8%), and thrombocytopenia (43.9% vs 29.7%). Although not recommended in international guidelines, some triplet regimens, including paclitaxel, ifosfamide, and nedaplatin [9, 10] or paclitaxel, cisplatin, and gemcitabine [29], are also used as second-line treatment regimens in real-world studies of bladder cancer in Japan. Both triplet regimens have been associated with development of grade 3–4 neutropenia [29, 30]. These studies highlight the need for more tolerable second-line therapies to treat patients with advanced UC.

The small sample size of the subgroup of Japanese patients in the KEYNOTE-045 study limits the comparison of the Japanese population with the overall study population and interpretation of the analyses presented herein. The study was not powered for formal efficacy comparisons in the Japanese subpopulation. Another limitation is that single-agent comparators, including vinflunine (not standard of care in Japan), may not be fully representative of second-line treatment options used in clinical practice in Japan. Regardless, the current analysis offers valuable information about the efficacy and safety of pembrolizumab in Japanese patients. Outcomes will be confirmed in the real-world clinical setting of patients treated in Japan.

In conclusion, the efficacy and safety profile of pembrolizumab as second-line treatment of advanced/metastatic UC in Japanese patients is consistent with the overall results of the international KEYNOTE-045 study. Pembrolizumab is an effective and well-tolerated treatment option that is recommended in international guidelines as the preferred second-line treatment for UC [23].

## Electronic supplementary material

Below is the link to the electronic supplementary material.

Supplementary file1 (PDF 278 kb)

## Data Availability

Merck Sharp & Dohme Corp., a subsidiary of Merck & Co., Inc., Kenilworth, NJ, USA’s, data sharing policy, including restrictions, is available at https://engagezone.msd.com/ds_documentation.php. Requests for access to the clinical study data can be submitted through the EngageZone site or via email to dataaccess@merck.com.
