# Supplementary material for: Pembrolizumab versus chemotherapy in recurrent, advanced urothelial cancer in Japanese patients: a subgroup analysis of the phase 3 KEYNOTE-045 trial
Source: Int J Clin Oncol. 2019 Nov 15;25(1):165–74. doi: 10.1007/s10147-019-01545-4 (PMC6946746; doi:10.1007/s10147-019-01545-4)

Online Resource

Article name: Pembrolizumab versus chemotherapy in recurrent, advanced urothelial cancer in Japanese patients: a subgroup analysis of the phase 3 KEYNOTE-045 trial

Journal name: *International Journal of Clinical Oncology*

Author names: Hiroyuki Nishiyama,[1]Yoshiaki Yamamoto,[2] Naoto Sassa,[3] Kazuo Nishimura,[4] Kiyohide Fujimoto,[5] Satoshi Fukasawa,[6] Minato Yokoyama,[7] Hideki Enokida,[8] Kenichi Takahashi,[9] Yoshinobu Tanaka,[9] Kentaro Imai,[10]Takashi Shimamoto,[9] Rodolfo Perini,[10] Tara Frenkl,[10] Dean Bajorin,[11] Joaquim Bellmunt[12,13]

Author affiliations:

[1]Yubinbango305-8576 Tsukuba, Ibaraki Prefecture Amakubo 2-chome address 1 1, University of Tsukuba Hospital, Tsukuba, Japan

[2]1 Chome-1-1 Minamikogush, Yamaguchi University Hospital, Ube, Japan

[3]65 Tsurumaicho, Showa Ward, Nagoya University Hospital, Nagoya, Japan

[4]3 Chome-1- 6 9 Otemae, Chuo Ward; Osaka International Cancer Institute, Osaka, Japan

[5]840 Shijocho, Nara Medical University Hospital, Kashihara, Japan

[6]666-2 Nitonacho, Chiba Cancer Center, Chiba, Japan

[7]1 Chome-5 4 5 Yushima, Medical Hospital, Tokyo Medical and Dental University, Tokyo, Japan

[8]8-35-1 Sakuragaoka, Kagoshima University Hospital, Kagoshima, Japan

[9]Kitanomaru Square, 1-13-12, Kudan Kita, MSD K.K., Tokyo, Japan

[10]2000 Galloping Hill Rd, Merck & Co., Inc., Kenilworth, NJ, USA

[11]1275 York Avenue, Memorial Sloan Kettering Cancer Center, New York, NY, USA

[12]Carrer del Dr. Aiguader, 88, PSMAR-IMIM Research Institute, Barcelona, Spain

[13]25 Shattuck St, Harvard Medical School, Boston, MA, USA

Corresponding author: Hiroyuki Nishiyama

Mailing address: Yubinbango305-8576 Tsukuba, Ibaraki Prefecture Amakubo 2-chome address 1 1, Tsukuba, Japan

Phone: +81 29-853-3900

Fax: +81 35-456-2556

Email: nishiuro@md.tsukuba.ac.jp

Fig. S1 KEYNOTE-045 Japanese subgroup analysis CONSORT patient disposition flow diagram. [a]Includes both clinical progression and disease progression. *AE* adverse event, *APaT* all patients as treated, *CR* complete response, *ITT* intent-to-treat, *PD* progressive disease

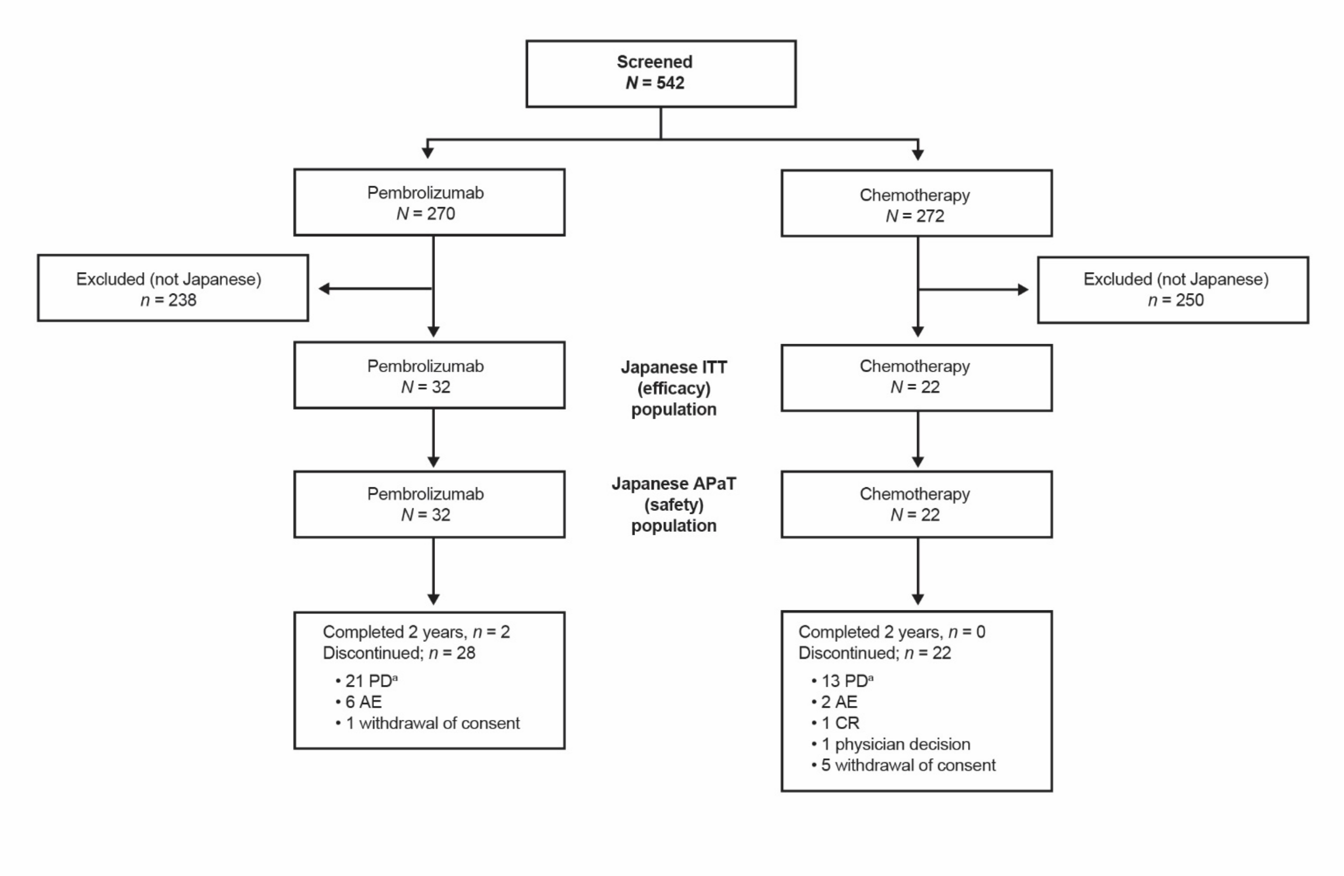

Supplement: Supplementary file 1 — Supplementary file1 (PDF 278 kb) [file 10147_2019_1545_MOESM1_ESM.pdf]
